# Supplementary material for: Acetic acid activates distinct taste pathways in Drosophila to elicit opposing, state-dependent feeding responses
Source: eLife. 2019 Jun 17;8:e47677. doi: 10.7554/eLife.47677 (PMC6579511; doi:10.7554/eLife.47677)
Supplement: Supplementary file 1. — Summary of all statistical results from this study. [file elife-47677-supp1.docx]

**Supplementary File 1: Summary of statistical results**

Summary of all statistical results from this study.

| **Experiment** | **Figure** | **Statistical Test** | **Test Main Results** | **Post-tests** | **Significant Post-test Results** |
| --- | --- | --- | --- | --- | --- |
| AA PER: comparisons between groups | 1A | 2-way repeated measures ANOVA | Group: p=0.0019  Concentration: p<0.0001  Interaction: p<0.0001 | Bonferroni | Fed vs. 1d starved: p<0.01 at 2.5% and 10%, p<0.001 at 5%  Fed vs. 2d starved: p<0.05 at 1%, p<0.001 at 2.5%, 5%, 10% |
| AA PER: comparisons within each group | 1A | 1-way repeated measures ANOVA | Fed: p=0.0312  1d starved: p<0.0001  2d starved: p<0.0001 | Dunnett (each AA conc. vs. 0% AA) | Fed: p<0.05 at 10%  1d starved: p<0.01 at 2.5%, 5%, 10%  2d starved: p<0.01 at 1%, 2.5%, 5%, 10% |
| Sucrose PER: comparisons between groups | 1B | 2-way repeated measures ANOVA | Group: p=0.0061  Concentration: p<0.0001  Interaction: p<0.0001 | Bonferroni | Fed vs. 1d starved: p<0.001 at 25 mM, 50 mM  Fed vs. 2d starved: p<0.05 at 5 mM, p<0.001 at 10 mM, 25 mM, 50 mM  1d vs. 2d starved: p<0.05 at 10 mM |
| AA + 300 mM sucrose PER: comparisons between groups | 1C | 2-way repeated measures ANOVA | Group: p<0.0001  Concentration: p<0.0001  Interaction: p<0.0001 | Bonferroni | Fed vs. 1d starved: p<0.001 at 1%, 5%, 10%  Fed vs. 2d starved: p<0.05 at 0%, p<0.001 at 1%, 5%, 10% |
| AA + 300 mM sucrose PER: comparisons within each group | 1C | 1-way repeated measures ANOVA | Fed: p=0.0010  1d starved: p=0.1121  2d starved: p=0.3839 | Dunnett (each AA conc. vs. 0% AA) | Fed: p<0.01 at 5%, 10% |
| AA + 50 mM sucrose PER: comparisons between groups | 1D | 2-way repeated measures ANOVA | Group: p<0.0001  Concentration: p<0.0001  Interaction: p=0.1039 | Bonferroni | Fed vs. 1d starved: p<0.001 at 0%, 1%, 5%, 10%  Fed vs. 2d starved: p<0.001 at 0%, 1%, 5%, 10%  1d vs. 2d starved: p<0.05 at 5%, 10% |
| AA + 50 mM sucrose PER: comparisons within each group | 1D | 1-way repeated measures ANOVA | Fed: p=0.0046  1d starved: p=0.0305  2d starved: p=0.0950 | Dunnett (each AA conc. vs. 0% AA) | Fed: p<0.05 at 5%, p<0.01 at 10%  1d starved: p<0.05 at 10% |
| AA PER (legs) | 1E | 1-way repeated measures ANOVA | p=0.0002 | Dunnett (each AA conc. vs. 0% AA) | p<0.01 at 1%, 2.5%, 5%, 10% |
| AA + 10 mM sucrose PER | 1-S1A | 1-way repeated measures ANOVA | p=0.0405 | Dunnett (each AA conc. vs. 0% AA) | p<0.05 at 5% |
| AA + 5 mM sucrose PER | 1-S1B | 1-way repeated measures ANOVA | p=0.0016 | Dunnett (each AA conc. vs. 0% AA) | p<0.01 at 5%, 10% |
| Bitter PER | 1-S2A | 1-way repeated measures ANOVA | Quinine: p=0.0005  Lobeline: p=0.1257 | Dunnett (each bitter conc. vs. H2O) | Quinine: p<0.05 at 0.1 mM, 10 mM |
| Bitter + sucrose PER | 1-S2B | 1-way repeated measures ANOVA | p<0.0001 | Bonferroni | p<0.001 for H2O vs. sucrose, sucrose vs. sucrose + quinine, sucrose vs. sucrose + lobeline, |
| HCl PER | 1-S3A | 1-way repeated measures ANOVA | p=0.0614 |  |  |
| Acetate PER | 1-S3B | 1-way repeated measures ANOVA | p=0.0663 |  |  |
| Carboxylic acid PER | 1-S3C | 1-way repeated measures ANOVA | p<0.0001 | Dunnett (each stim vs H2O) | Citric acid: p<0.05 at 100 mM, 1000 mM  Propionic acid: p<0.01 at 5%, 10% |
| Olfactory ablation (2d starved) | 2A | 2-way repeated measures ANOVA | Group: p=0.3031  Concentration: p<0.0001  Interaction: p=0.7107 |  |  |
| Olfactory ablation (fed, AA alone) | 2B | 2-way repeated measures ANOVA | Group: p=0.4810  Concentration: p=0.8841  Interaction: p=0.3514 |  |  |
| Olfactory ablation (fed, AA + sucrose): comparisons between groups | 2C | 2-way repeated measures ANOVA | Group: p=0.0032  Concentration: p=0.0001  Interaction: p=0.4131 | Bonferroni | p<0.05 at 0%, p<0.01 at 5% |
| Olfactory ablation (fed, AA + sucrose): comparisons within each group | 2C | 1-way repeated measures ANOVA | Ctrl: p=0.0057  Olfactory ablation: p=0.0123 | Dunnett (each AA conc vs. 0% AA) | Ctrl: p<0.01 at 5%, 10%  Olfactory ablation: p<0.05 at 5%, p<0.01 at 10% |
| *poxn* mutant PER | 2D | 2-way repeated measures ANOVA | Genotype: p<0.0001  Concentration: p<0.0001  Interaction: p<0.0001 | Bonferroni (*poxn* mutant vs. each ctrl) | p<0.001 at 5%, 10% for mutant vs. each ctrl |
| Sugar neuron silencing: sucrose PER | 3A | 2-way repeated measures ANOVA | Genotype: p<0.0001  Concentration: p<0.0001  Interaction: p<0.0001 | Bonferroni (experimental vs. each ctrl) | Expt vs. *Gal4/+*: p<0.01 at 5 mM, p<0.001 at 10 mM, 25 mM, 50mM  Expt vs. *UAS/+*: p<0.001 at 10 mM, 25 mM, 50 mM |
| Sugar neuron silencing: AA PER | 3B | 2-way repeated measures ANOVA | Genotype: p=0.0015  Concentration: p<0.0001  Interaction: p<0.0001 | Bonferroni (experimental vs. each ctrl) | Expt vs. *Gal4/+*: p<0.05 at 2.5%, p<0.01 at 5%, p<0.001 at 10%  Expt vs. *UAS/+*: p<0.001 at 5%, 10% |
| *∆8Grs* mutant: sucrose PER | 3C | 2-way repeated measures ANOVA | Genotype: p<0.0001  Concentration: p<0.0001  Interaction: p<0.0001 | Bonferroni (ctrl vs. mutant) | p<0.001 at all nonzero sucrose concentrations |
| *∆8Grs* mutant: AA PER | 3D | 2-way repeated measures ANOVA | Genotype: p=0.0014  Concentration: p<0.0001  Interaction: p<0.0001 | Bonferroni (ctrl vs. mutant) | p<0.001 at all nonzero AA concentrations |
| *norpA* RNAi: fatty acid PER | 3E | 2-way repeated measures ANOVA | Genotype: p=0.0004  Concentration: p=0.0003  Interaction: p=0.0026 | Bonferroni (experimental vs. each ctrl) | Expt vs. *Gal4/+* or *UAS/+*: p<0.001 at both nonzero concentrations |
| *norpA* RNAi: sucrose PER | 3F | 2-way repeated measures ANOVA | Genotype: p=0.4889  Concentration: p<0.0001  Interaction: p=0.7986 |  |  |
| *norpA* RNAi: AA PER | 3G | 2-way repeated measures ANOVA | Genotype: p=0.8405  Concentration: p<0.0001  Interaction: p=0.9958 |  |  |
| Bitter neuron silencing: AA + sucrose PER, comparisons between groups | 4A | 2-way repeated measures ANOVA | Genotype: p=0.0141  Concentration: p<0.0001  Interaction: p=0.0002 | Bonferroni (experimental vs. each ctrl) | Expt vs. *Gal4/+*: p<0.001 at 10%  Expt vs. *UAS/+*: p<0.01 at 2.5%, 5%, p<0.001 at 10% |
| Bitter neuron silencing: AA + sucrose PER, comparisons within each group | 4A | 1-way repeated measures ANOVA | Expt: p=0.0775  Gal4+: p<0.0001  UAS/+: p=0.0003 | Dunnett (each AA conc vs. 0% AA) | *Gal4/+*: p<0.01 at 2.5%, 5%, 10%  *UAS/+*: p<0.01 at 2.5%, 5%, 10% |
| Bitter neuron silencing: AA PER (fed) | 4B | 2-way repeated measures ANOVA | Genotype: p=0.0006  Concentration: p<0.0001  Interaction: p<0.0001 | Bonferroni (experimental vs. each ctrl) | Expt vs. *Gal4/+* or *UAS/+*: p<0.001 at all nonzero concentrations |
| Bitter neuron silencing: AA PER (starved) | 4C | 2-way repeated measures ANOVA | Genotype: p=0.0194  Concentration: p<0.0001  Interaction: p=0.0051 | Bonferroni (experimental vs. each ctrl) | Expt vs. *Gal4/+*: p<0.01 at 5%, p<0.001 at 2.5%  Expt vs. *UAS/+*: p<0.05 at 5%, p<0.001 at 2.5% |
| *Gal4/+* ctrl: AA PER (fed vs. starved) | 4D | 2-way ANOVA | Group: p<0.0001  Concentration: p<0.0001  Interaction: p=0.0054 | Bonferroni | p<0.05 at 2.5%, p<0.01 at 1%, p<0.001 at 5%, 10% |
| *UAS/+* ctrl: AA PER (fed vs. starved) | 4E | 2-way ANOVA | Group: p<0.0001  Concentration: p<0.0001  Interaction: p<0.0001 | Bonferroni | p<0.01 at 1%, p<0.001 at 2.5%, 5%, 10% |
| *Gal4/UAS* expt: AA PER (fed vs. starved) | 4F | 2-way ANOVA | Group: p=0.0090  Concentration: p<0.0001  Interaction: p=0.0021 | Bonferroni | p<0.05 at 2.5%, 5% |
| Bitter neuron silencing: quinine PER suppression | 4-S1A | 2-way repeated measures ANOVA | Genotype: p<0.0001  Concentration: p<0.0001  Interaction: p<0.0001 | Bonferroni (experimental vs. each ctrl) | Expt vs *Gal4/+*: p<0.001 at 10 mM  Expt vs. *UAS/+*: p<0.05 at 1 mM, p<0.001 at 10 mM |
| Bitter neuron silencing: lobeline PER suppression | 4-S1B | 2-way repeated measures ANOVA | Genotype: p<0.0001  Concentration: p<0.0001  Interaction: p<0.0001 | Bonferroni (experimental vs. each ctrl) | Expt vs *Gal4/+* or *UAS/+*: p<0.001 at 1 mM, 5 mM |
| Bitter neuron silencing: sucrose PER (fed) | 4-S1C | 2-way repeated measures ANOVA | Genotype: p=0.2952  Concentration: p<0.0001  Interaction: p=0.9881 |  |  |
| Bitter neuron silencing: sucrose PER (starved) | 4-S1D | 2-way repeated measures ANOVA | Genotype: p=0.9687  Concentration: p<0.0001  Interaction: p=0.7825 |  |  |
| *IR25a* mutants: AA PER (starved) | 4-S2A | 2-way repeated measures ANOVA | Genotype: p=0.0581  Concentration: p<0.0001  Interaction: p=0.0080 | Bonferroni (ctrl vs. each mutant) | *IR25a^2^*: p<0.05 at 5%, p<0.01 at 2.5%  *IR25a^1^/IR25a^2^*: p<0.01 at 2.5%, 5% |
| *IR25a* mutants: AA PER (fed) | 4-S2B | 2-way repeated measures ANOVA | Genotype: p=0.0037  Concentration: p<0.0001  Interaction: p=0.0037 | Bonferroni (ctrl vs. each mutant) | *IR25a^2^*: p<0.01 at 10%, p<0.001 at 5%  *IR25a^1^/IR25a^2^*: p<0.05 at 1%, p<0.01 at 10%, p<0.001 at 5% |
| *IR25a* mutants: AA + suc PER (comparisons between groups) | 4-S2C | 2-way repeated measures ANOVA | Genotype: p=0.2504  Concentration: p<0.0001  Interaction: p=0.0973 |  |  |
| *IR25a* mutants: AA + suc PER (comparisons within each group) | 4-S2C | 1-way repeated measures ANOVA | *w^1118^*: p=0.3351  *IR25a^2^*: p<0.0001  *IR25a^1^/IR25a^2^*: p=0.0307 | Dunnett (each AA conc vs. 0% AA) | *IR25a^2^*: p<0.01 at 5%, 10% |
| *IR76b* mutants: AA PER (starved) | 4-S2D | 2-way repeated measures ANOVA | Genotype: p=0.1856  Concentration: p<0.0001  Interaction: p=0.1065 |  |  |
| *IR76b* mutants: AA PER (fed) | 4-S2E | 2-way repeated measures ANOVA | Genotype: p=0.0258  Concentration: p<0.0001  Interaction: p=0.1234 | Bonferroni (ctrl vs. each mutant) | *IR76b^1^/IR76b^2^*: p<0.05 at 5%, p<0.01 at 10% |
| *IR76b* mutants: AA + suc PER (comparisons between groups) | 4-S2F | 2-way repeated measures ANOVA | Genotype: p=0.4902  Concentration: p<0.0001  Interaction: p=0.0108 | Bonferroni (ctrl vs. each mutant) | *IR76b^2^*: p<0.05 at 5% |
| *IR76b* mutants: AA + suc PER (comparisons within each group) | 4-S2F | 1-way repeated measures ANOVA | *w^1118^*: p=0.0697  *IR76b^1^*: p=0.0337  *IR76b^2^*: p=0.0050  *IR76b^1^/IR76b^2^*: p=0.0007 | Dunnett (each AA conc vs. 0% AA) | *IR76b^1^*: p<0.05 at 1%  *IR76b^2^*: p<0.05 at 10%, p<0.01 at 1%, 5%  *IR76b^1^/IR76b^2^*: p<0.05 at 5%, p<0.01 at 1% |
| *IR76b^1^* mutant vs. het ctrl: AA + suc PER (comparisons between groups) | 4-S2G | 2-way repeated measures ANOVA | Genotype: p=0.2757  Concentration: p<0.0001  Interaction: p=0.1665 |  |  |
| *IR76b^1^* mutant vs. het ctrl: AA + suc PER (comparisons within each group) | 4-S2G | 1-way repeated measures ANOVA | *IR76b^1^*: p=0.0008  *IR76b^1^/+*: p=0.0890 | Dunnett (each AA conc vs. 0% AA) | *IR76b^1^*: p<0.05 at 10%, p<0.01 at 1%, 5% |
| *IR76b^2^* mutant vs. het ctrl: AA + suc PER (comparisons between groups) | 4-S2H | 2-way repeated measures ANOVA | Genotype: p=0.7972  Concentration: p<0.0001  Interaction: p=0.0005 | Bonferroni | p<0.05 at 0% |
| *IR76b^2^* mutant vs. het ctrl: AA + suc PER (comparisons within each group) | 4-S2H | 1-way repeated measures ANOVA | *IR76b^2^*: p=0.0001  *IR76b^2^/+*: p=0.0002 | Dunnett (each AA conc vs. 0% AA) | *IR76b^2^*: p<0.01 at 1%, 5%, 10%  *IR76b^2^/+*: p<0.01 at 1%, 5% |
| Sugar neuron responses to sucrose: peak ∆F/F_0_ | 5D | 2-way ANOVA | Group: p=0.0633  Stimulus: p<0.0001  Interaction: p=0.4184 | Bonferroni (each stimulus vs. H2O) | Fed: p<0.001 at 500 mM suc  Starved: p<0.001 at 500 mM suc |
| Sugar neuron responses to sucrose: integrated ∆F/F_0_ | – | 2-way ANOVA | Group: p=0.0364  Stimulus: p<0.0001  Interaction: p=0.3114 | Bonferroni (each stimulus vs. H2O; fed vs. starved) | Fed: p<0.001 at 500 mM  Starved: p<0.001 at 500 mM  Fed vs. starved: p<0.05 at 500 mM |
| Sugar neuron responses to AA | 5D | 2-way ANOVA | Group: p=0.0003  Stimulus: p<0.0001  Interaction: p=0.4331 | Bonferroni (each stimulus vs. H2O; fed vs. starved) | Fed: p<0.001 at 1%, 5%  Starved: p<0.001 at 1%, 5%  Fed vs. starved: p<0.01 at 1% |
| Bitter neuron responses to lobeline: peak ∆F/F_0_ | 5H | 2-way ANOVA | Group: p=0.3203  Stimulus: p<0.0001  Interaction: p=.3486 | Bonferroni (each stimulus vs. H2O) | Fed: p<0.001 at 0.1 mM, 1 mM  Starved: p<0.001 at 0.1 mM, 1 mM |
| Bitter neuron responses to lobeline: integrated ∆F/F_0_ | – | 2-way ANOVA | Group: p=0.2446  Stimulus: p<0.0001  Interaction: p=.2259 | Bonferroni (each stimulus vs. H2O) | Fed: p<0.001 at 0.1 mM, 1 mM  Starved: p<0.001 at 0.1 mM, 1 mM |
| Bitter neuron responses to AA | 5H | 2-way ANOVA | Group: p=0.0027  Stimulus: p<0.0001  Interaction: p<0.0001 | Bonferroni (each stimulus vs. H2O; fed vs. starved) | Fed: p<0.05 at 1%, p<0.001 at 5%  Starved: p<0.01 at 5%, p<0.001 at 1%  Fed vs. starved: p<0.001 at 1% |
| Sugar neuron dose response (all trials) | 5-S3A | 1-way ANOVA | p<0.0001 | Dunnett (each stimulus vs. H2O) | p<0.05 for 5% AA, p<0.01 for 1% AA, sucrose |
| Sugar neuron dose response (1st trials excluded) | 5-S3A | 1-way ANOVA | p<0.0001 | Dunnett (each stimulus vs. H2O) | p<0.05 for 0.1% AA, 5% AA, p<0.01 for 1% AA, sucrose |
| Bitter neuron dose response (all trials) | 5-S3B | 1-way ANOVA | p<0.0001 | Dunnett (each stimulus vs. H2O) | p<0.05 for 0.1% AA, 1% AA, 5% AA, p<0.01 for 0.01% AA, denatonium |
| Bitter neuron dose response (1st trials excluded) | 5-S3B | 1-way ANOVA | p<0.0001 | Dunnett (each stimulus vs. H2O) | p<0.05 for 0.1% AA, 1% AA, p<0.01 for 0.01% AA, 5% AA, denatonium |
| *∆8Grs* mutant imaging | 5-S4B | 2-way ANOVA | Genotype: p=0.0112  Stimulus: p<0.0001  Interaction: p<0.0001 | Bonferroni (each stimulus vs. H2O; mutant vs. ctrl) | Ctrl: p<0.05 for 5% AA, p<0.001 for sucrose  Mutant vs. ctrl: p<0.001 for sucrose |
| Bitter neuron subsets imaging | 5-S5E | 1-way ANOVA | p<0.0001 for all subsets | Dunnett (each stimulus vs. H2O) | *Gr98d-Gal4*: p<0.01 for lobeline, caffeine  *Gr22f-Gal4*: p<0.01 for lobeline, caffeine, 1% AA  *Gr59c-Gal4*: p<0.01 for lobeline  *Gr47a-Gal4*: p<0.01 for lobeine |
| Water neuron imaging (± PEG) | 5-S6D | 2-way ANOVA | Presence of PEG: p<0.0001  Stimulus: p<0.0001  Interaction: p=0.0005 | Bonferroni (each stimulus vs. H2O; PEG vs. no PEG) | No PEG: p<0.01 at 1%, p<0.001 at 5%  PEG vs. no PEG: p<0.001 for H2O, 1% AA |
| *Gr64f-Gal4/ UAS-GCaMP6f* AA PER | 5-S7A | 2-way repeated measures ANOVA | Group: p=0.0005  Concentration: p<0.0001  Interaction: p=0.0004 | Bonferroni (comparing groups) | Fed vs. 1d starved: p<0.05 at 5%, p<0.01 at 2.5%, 10%  Fed vs. 2d starved: p<0.01 at 1%, p<0.001 at 2.5%, 5%, 10%  1d vs. 2d starved: p<0.05 at 2.5% |
| *Gr64f-Gal4/ UAS-GCaMP6f* sucrose PER | 5-S7B | 2-way ANOVA | Group: p<0.0001  Concentration: p<0.0001  Interaction: p<0.0001 | Bonferroni (comparing groups) | Fed vs. 1d starved: p<0.05 at 25 mM, p<0.01 at 50 mM, p<0.001 at 10 mM  Fed vs. 2d starved: p<0.001 at 5 mM, 10 mM, 25 mM, 50 mM  1d vs. 2d starved: p<0.001 at 5 mM, 10 mM |
| *Gr66a-Gal4 /UAS-GCaMP6f* AA PER | 5-S7C | 2-way repeated measures ANOVA | Group: p=0.0024  Concentration: p<0.0001  Interaction: p=0.0014 | Bonferroni (comparing groups) | Fed vs. 1d starved: p<0.01 at 10%  Fed vs. 2d starved: p<0.05 at 1%, p<0.001 at 2.5%, 5%, 10%  1d vs. 2d starved: p<0.05 at 5%, p<0.01 at 2.5% |
| *Gr66a-Gal4/ UAS-GCaMP6f* sucrose PER | 5-S7D | 2-way ANOVA | Group: p<0.0001  Concentration: p<0.0001  Interaction: p=0.0066 | Bonferroni (comparing groups) | Fed vs. 1d starved: p<0.05 at 25 mM  Fed vs. 2d starved: p<0.01 at 50 mM, p<0.001 at 10 mM, 25 mM  1d vs. 2d starved: p<0.05 at 10 mM |
